# Emotional and behavioral problems in the offspring of parents with schizophrenia or bipolar disorder: a controlled comparative study

**DOI:** 10.3389/fpsyt.2026.1847199

**Published:** 2026-09-09

**Authors:** Umut Balatacı, Bari Ay, Rıfat Tarhan, Ayşegül Ay

**Affiliations:** 1 Department of Child and Adolescent Psychiatry, Biruni University Faculty of Medicine, İstanbul, Türkiye; 2 Clinic of Child and Adolescent Psychiatry, Private Clinic, Karabük, Türkiye; 3 Department of Psychiatry, Karabük University Faculty of Medicine, Karabük, Türkiye; 4 Operating Room Services Program, Department of Medical Services and Techniques, Vocational School of Health Services, Karabük University, Karabük, Türkiye

**Keywords:** bipolar disorder, child behavior checklist, child psychiatry, offspring, schizophrenia

## Abstract

**Objective:**

This study aimed to compare emotional and behavioral problems in the offspring of parents with schizophrenia, the offspring of parents with bipolar disorder, and healthy controls using the Child Behavior Checklist for ages 6–18 years (CBCL/6–18).

**Methods:**

This cross-sectional controlled study was conducted in the Child and Adolescent Psychiatry Outpatient Clinic of Karabük Training and Research Hospital. The sample included 62 children and adolescents aged 6–18 years: 21 offspring of parents with schizophrenia, 20 offspring of parents with bipolar disorder, and 21 healthy controls. Parental diagnoses were established according to the criteria in the Diagnostic and Statistical Manual of Mental Disorders, Fifth Edition (DSM-5) and confirmed with the Structured Clinical Interview for DSM-5. Emotional and behavioral problems were assessed using CBCL/6–18 T-scores. Group comparisons were performed using one-way analysis of variance and chi-square tests, with Bonferroni correction applied for multiple CBCL comparisons. Multivariable linear regression analysis was used to identify factors independently associated with total problems scores.

**Results:**

The groups did not differ significantly in age, sex, or educational status. Compared with healthy controls, both clinical groups had higher rates of previous child psychiatry visits and less favorable family-related sociodemographic characteristics. After Bonferroni correction, significant group differences remained for withdrawn/depressed, anxious/depressed, internalizing problems, aggressive behavior, externalizing problems, social problems, attention problems, and total problems. In these domains, both offspring groups scored significantly higher than controls, whereas the schizophrenia and bipolar disorder groups did not significantly differ from one another. In adjusted regression analysis, both schizophrenia-group status and bipolar-disorder-group status remained independently associated with higher total problems scores.

**Conclusion:**

Offspring of parents with schizophrenia or bipolar disorder show elevated emotional and behavioral problems compared with healthy controls, supporting the importance of early screening and family-oriented preventive interventions.

## Introduction

Schizophrenia and bipolar disorder are severe psychiatric disorders with substantial heritable liability, and increasing evidence suggests that they also share part of their genetic architecture rather than representing fully distinct familial risk pathways. Offspring of parents with severe mental illness have a markedly elevated risk of later psychopathology, and meta-analytic data indicate that nearly one in three may develop a severe mental illness by early adulthood (1–3). Family high-risk studies further show that this vulnerability is not limited to the eventual emergence of psychotic or bipolar syndromes; instead, early difficulties often appear as broader emotional, behavioral, cognitive, and social problems during childhood and adolescence (3–5).

Prior work has shown that children at familial high risk for schizophrenia or bipolar disorder exhibit more psychopathology than controls from an early age. In the Danish High Risk and Resilience Study, 7-year-old children with familial high risk for schizophrenia spectrum psychosis or bipolar disorder showed higher levels of psychopathology than population-based controls, with elevated parent- and teacher-rated difficulties across several domains (6, 7). Similarly, longitudinal and cross-disorder studies have reported that adolescent offspring of parents with schizophrenia or bipolar disorder have higher rates of categorical and dimensional psychopathology, while offspring of parents with bipolar disorder have also been shown to display increased rates of attention-deficit/hyperactivity disorder (ADHD), disruptive symptoms, mood symptoms, and broader functional difficulties even in preschool and school-age samples (8–10). Offspring of parents with schizophrenia are likewise at increased risk for schizophrenia-spectrum and other psychiatric conditions during youth, supporting the view that early vulnerability may be clinically detectable before the onset of major syndromic illness (11).

From a clinical perspective, these findings make the systematic assessment of behavioral and emotional problems in high-risk offspring especially important. The Child Behavior Checklist is one of the most widely studied parent-report instruments for evaluating emotional and behavioral problems in children and adolescents, with broad support for its reliability and validity across clinical populations (12). However, although the literature supports elevated psychopathology in offspring of parents with schizophrenia and bipolar disorder, relatively few studies have directly compared these two familial high-risk groups with healthy controls within the same design using dimensional behavioral measures (5, 8). Therefore, the present study aimed to compare emotional and behavioral problems in children of parents with schizophrenia, children of parents with bipolar disorder, and healthy controls, and to clarify whether these high-risk groups show a broader burden of internalizing, externalizing, and total problem behaviors.

## Materials and methods

### Study design and setting

This cross-sectional controlled study was conducted at the Child and Adolescent Psychiatry Outpatient Clinic of Karabük Training and Research Hospital. The study was designed to compare emotional and behavioral problems among children of parents with schizophrenia, children of parents with bipolar disorder, and healthy controls. According to the ethics application, eligible families were identified through the adult psychiatry outpatient services of the same institution, and children who met study criteria were referred for child and adolescent psychiatric evaluation.

### Ethical approval and research standards

Ethical approval was obtained from the Karabük University Non-Interventional Clinical Research Ethics Committee on 28 July 2025 (decision no. 2025/2430). The committee reviewed the protocol and approved the study unanimously as ethically appropriate. The study was conducted in accordance with the Declaration of Helsinki, which the World Medical Association identifies as a statement of ethical principles for medical research involving human participants (13). Before participation, all parents or legal guardians were informed in detail about the aim and procedures of the study, the voluntary nature of participation, confidentiality protections, and the right to withdraw at any time without any negative effect on clinical care. Written informed consent was obtained from all participating families prior to data collection. The ethics application also stated that participants’ personal data would be kept confidential and that any study-related procedures, if required, would be carried out under physician and/or healthcare personnel supervision.

### Participants

The study population consisted of children and adolescents aged 6–18 years. The schizophrenia group included offspring of parents who were being followed in the adult psychiatry outpatient clinic with a diagnosis of schizophrenia, whereas the bipolar disorder group included offspring of parents with bipolar disorder. In both clinical groups, the parental diagnosis of schizophrenia or bipolar disorder was established according to the criteria in the Diagnostic and Statistical Manual of Mental Disorders, Fifth Edition (DSM-5) and confirmed by an adult psychiatrist using the Structured Clinical Interview for DSM-5 (SCID-5). The healthy control group consisted of children of parents who had presented to the adult psychiatry outpatient clinic for various reasons but were considered psychiatrically healthy at the time of the study following evaluation by an adult psychiatrist. The absence of schizophrenia spectrum disorder, bipolar disorder, and other major psychiatric disorders was confirmed according to DSM-5 criteria using the SCID-5. Two parents in the control group had a past history of adjustment disorder; however, neither had a current psychiatric disorder at the time of the study evaluation. All children included in the study were evaluated by two child and adolescent psychiatrists.

### Inclusion and exclusion criteria

The inclusion criteria were as follows: age 6–18 years; at least one parent with a diagnosis of schizophrenia or bipolar disorder for the clinical groups; a parent or legal guardian capable of providing reliable developmental and behavioral information; and provision of written informed consent. In the control group, an additional inclusion criterion was the absence of major psychiatric illness in the parents on adult psychiatric evaluation.

The exclusion criteria were derived from the ethics protocol and detailed in accordance with the familial high-risk literature to ensure valid behavioral assessment. Children were excluded if they had any physical, neurological, developmental, or psychiatric condition severe enough to interfere with comprehension, cooperation, or reliable evaluation; if informed consent was not obtained; or if the parent informant was unable to complete the study procedures reliably. In practical terms, these conditions included moderate-to-severe intellectual disability, autism spectrum disorder with marked communication impairment, major neurological disease, severe sensory impairment, or acute psychiatric/medical instability that could compromise assessment validity. For the healthy control group, any current or lifetime major psychiatric disorder in either parent also led to exclusion. Familial high-risk studies similarly emphasize careful diagnostic characterization of parents and the exclusion of factors that may compromise the validity of offspring assessment.

### Sample size and power analysis

An *a priori* power analysis was performed using G*Power version 3.1 (Heinrich Heine University Düsseldorf, Düsseldorf, Germany) to estimate the minimum sample size required for comparisons across the three study groups (14). Based on a previous familial high-risk offspring study (5) showing meaningful differences in dimensional psychopathology between offspring of parents with schizophrenia spectrum disorder, bipolar disorder, and healthy controls, the analysis was conducted for a one-way ANOVA with three groups, α = 0.05, 80% power, and an assumed effect size of f = 0.41. The minimum required sample size was 61 participants. The final sample comprised 62 participants (21 in the schizophrenia group, 20 in the bipolar disorder group, and 21 in the healthy control group), indicating that the study had an adequate sample size for the primary group comparisons.

### Clinical evaluation and data collection

After eligibility screening, the parent serving as the primary informant completed a sociodemographic data form developed for the study. This form included the child’s age, sex, school attendance, prior child psychiatry visit, family structure, parental marital status, income level, parental employment status, educational level, and history of parental psychiatric disorder. As specified in the ethics application, the main study instrument was then administered to the parent informant.

### Child behavior checklist

Behavioral and emotional problems were assessed using the Child Behavior Checklist for ages 6–18 years (CBCL/6–18), a parent-report measure developed by Achenbach and Rescorla for assessing behavioral and emotional problems in children and adolescents (15). The Turkish adaptation and standardization of the CBCL/6–18 were reported by Erol, Arslan, and Akçakın (16). The CBCL provides syndrome-scale scores and broad-band scores for internalizing problems, externalizing problems, and total problems (16). Parents rate each item on a 3-point Likert scale ranging from 0 to 2, with higher scores indicating greater symptom burden. Standardized T-scores obtained from the CBCL/6–18 were used in the analyses to evaluate the severity of emotional and behavioral problems in the participants.

### Statistical analysis

All statistical analyses were performed using IBM SPSS Statistics for Windows, version 27.0 (IBM Corp., Armonk, NY, USA). Continuous variables were expressed as mean ± standard deviation, and categorical variables were presented as number and percentage. The normality of continuous variables was assessed using the Kolmogorov–Smirnov test. Comparisons of continuous variables among the three groups were performed using one-way analysis of variance (ANOVA), followed by Tukey’s honestly significant difference (HSD) test for post-hoc pairwise comparisons when the overall group effect was significant. Because 11 CBCL/6–18 domain and broadband scores were compared across the three groups, Bonferroni correction was applied to control the family-wise type I error rate. Accordingly, the conventional significance level of 0.05 was divided by 11, resulting in a corrected significance threshold of p < 0.0045 (0.05/11 = 0.004545). For CBCL/6–18 outcomes meeting this corrected threshold in the overall ANOVA, Tukey’s HSD test was used to examine pairwise differences among the three groups. Categorical variables were compared using Pearson’s chi-square test or Fisher’s exact test, as appropriate. For all other analyses, a two-tailed p-value of less than 0.05 was considered statistically significant. Multivariable linear regression analysis was used to identify factors independently associated with the CBCL/6–18 total problems T-score, with diagnostic group as the main predictor and age, sex, previous child psychiatry visit, and parental marital status entered as covariates. The model assumptions evaluated before interpretation included linearity, normality of residuals, homoscedasticity, and multicollinearity. Multicollinearity was assessed using the variance inflation factor (VIF), and no problematic multicollinearity was observed.

## Results

**Table 1** presents the sociodemographic and clinical characteristics of the participants. The mean age of the children was 11.3 ± 3.4 years in the schizophrenia group, 11.8 ± 3.1 years in the bipolar disorder group, and 11.6 ± 3.2 years in the healthy control group, with no significant difference among the groups (p = 0.88). Sex distribution was also similar, with female participants comprising 47.6%, 50.0%, and 47.6% of the groups, respectively (p = 0.93). Educational status did not differ significantly between groups (p = 0.62). However, a previous child psychiatry visit was significantly more frequent in the schizophrenia and bipolar disorder groups than in healthy controls (38.1%, 35.0%, and 9.5%, respectively; p = 0.032). Parental marital status differed significantly across groups, with parents living together in 52.4% of the schizophrenia group, 65.0% of the bipolar disorder group, and 90.5% of the control group (p = 0.018). Poor income level was more common in the schizophrenia and bipolar disorder groups (42.9% and 30.0%, respectively) than in the control group (9.5%) (p = 0.011). Maternal psychiatric disorder was present in 33.3% of the schizophrenia group and 40.0% of the bipolar disorder group, compared with 4.8% in controls (p = 0.006), while paternal psychiatric disorder was observed in 52.4%, 45.0%, and 4.8% of the groups, respectively (p < 0.001). In addition, significant between-group differences were observed in maternal employment status (p = 0.027), maternal educational level (p = 0.049), paternal employment status (p = 0.021), and paternal educational level (p = 0.038). Overall, the schizophrenia and bipolar disorder groups showed a less favorable family-related sociodemographic profile compared with healthy controls.

**Table 1:** Sociodemographic and clinical characteristics of the study groups.

Variable	Schizophrenia (n=21)	Bipolar disorder (n=20)	Healthy controls (n=21)	p-value
Child characteristics				
Age, years	11.3 ± 3.4	11.8 ± 3.1	11.6 ± 3.2	0.886
Sex				0.935
Female	10 (47.6%)	10 (50.0%)	10 (47.6%)	
Male	11 (52.4%)	10 (50.0%)	11 (52.4%)	
Educational status				0.624
Not attending school	2 (9.5%)	1 (5.0%)	1 (4.8%)	
Primary school	8 (38.1%)	7 (35.0%)	7 (33.3%)	
Middle school	5 (23.8%)	5 (25.0%)	6 (28.6%)	
High school	6 (28.6%)	7 (35.0%)	7 (33.3%)	
Previous child psychiatry visit	8 (38.1%)^a^	7 (35.0%)^a^	2 (9.5%)^b^	**0.032**
Family characteristics				
Parental marital status				**0.018**
Living together	11 (52.4%)^a^	13 (65.0%)^ab^	19 (90.5%)^b^	
Separated	10 (47.6%)^a^	7 (35.0%)^ab^	2 (9.5%)^b^	
Income level				**0.011**
Poor	9 (42.9%)^a^	6 (30.0%)^ab^	2 (9.5%)^b^	
Moderate	10 (47.6%)	11 (55.0%)	12 (57.1%)	
Good	2 (9.5%)^a^	3 (15.0%)^ab^	7 (33.3%)^b^	
Parental characteristics				
Mother’s age, years	38.9 ± 5.8	37.6 ± 5.2	39.8 ± 4.9	0.410
Mother’s employment status				**0.027**
Employed	5 (23.8%)^a^	7 (35.0%)^ab^	11 (52.4%)^b^	
Unemployed	16 (76.2%)^a^	13 (65.0%)^ab^	10 (47.6%)^b^	
Mother’s educational level				**0.049**
Primary school	9 (42.9%)^a^	6 (30.0%)^ab^	3 (14.3%)^b^	
Middle school	5 (23.8%)	4 (20.0%)	4 (19.0%)	
High school	5 (23.8%)	6 (30.0%)	7 (33.3%)	
University	2 (9.5%)^a^	4 (20.0%)^ab^	7 (33.3%)^b^	
Maternal psychiatric disorder	7 (33.3%)^a^	8 (40.0%)^a^	1 (4.8%)^b^	**0.006**
Father’s age, years	42.1 ± 6.3	41.3 ± 5.9	41.9 ± 5.4	0.892
Father’s employment status				**0.021**
Employed	13 (61.9%)^a^	15 (75.0%)^ab^	20 (95.2%)^b^	
Unemployed	8 (38.1%)^a^	5 (25.0%)^ab^	1 (4.8%)^b^	
Father’s educational level				**0.038**
Primary school	8 (38.1%)^a^	6 (30.0%)^ab^	3 (14.3%)^b^	
Middle school	5 (23.8%)	4 (20.0%)	4 (19.0%)	
High school	6 (28.6%)	7 (35.0%)	8 (38.1%)	
University	2 (9.5%)^a^	3 (15.0%)^ab^	6 (28.6%)^b^	
Paternal psychiatric disorder	11 (52.4%)^a^	9 (45.0%)^a^	1 (4.8%)^b^	**< 0.001**

Values are presented as mean ± SD or n (%), as appropriate. p-values were obtained using one-way ANOVA for continuous variables and Pearson’s chi-square test or Fisher’s exact test for categorical variables, as appropriate. Groups sharing the same superscript letter are not significantly different, whereas groups with different superscript letters differ significantly in post-hoc pairwise comparisons. Bold p values indicate statistical significance (p < 0.05). In the healthy control group, two parents had a history of adjustment disorder but had no current psychiatric disorder at the time of the study evaluation.

**Table 2** presents the comparison of CBCL/6–18 T-scores among the three groups. All 11 CBCL outcomes showed significant overall group differences at the conventional p < 0.05 level. After Bonferroni correction for 11 comparisons (p < 0.0045), the differences remained significant for withdrawn/depressed, anxious/depressed, internalizing problems, aggressive behavior, externalizing problems, social problems, attention problems, and total problems. For these outcomes, both offspring groups generally had higher scores than healthy controls, whereas no significant differences were observed between the schizophrenia and bipolar disorder groups. Somatic complaints, thought problems, and rule-breaking behavior did not remain significant after Bonferroni correction.

**Table 2:** Comparison of CBCL/6–18 T-scores among the groups.

CBCL/6–18 scale	Schizophrenia (n=21)	Bipolar disorder (n=20)	Healthy controls (n=21)	p-value
Withdrawn/depressed	61.8 ± 8.7^a^	58.6 ± 8.1^a^	52.4 ± 6.9^b^	**< 0.001**
Somatic complaints	58.2 ± 7.4^a^	56.7 ± 7.2^a^	52.1 ± 5.8^b^	0.008
Anxious/depressed	64.5 ± 9.1^a^	61.9 ± 8.6^a^	54.0 ± 7.1^b^	**< 0.001**
Internalizing problems	63.7 ± 8.8^a^	61.2 ± 8.3^a^	53.8 ± 6.7^b^	**< 0.001**
Rule-breaking behavior	59.4 ± 7.9^a^	56.2 ± 7.0^ab^	52.8 ± 5.9^b^	0.012
Aggressive behavior	62.3 ± 8.6^a^	59.8 ± 8.2^a^	53.7 ± 6.3^b^	**0.001**
Externalizing problems	61.5 ± 8.1^a^	58.4 ± 7.7^a^	53.1 ± 6.4^b^	**0.002**
Social problems	60.8 ± 7.5^a^	58.9 ± 7.3^a^	53.6 ± 5.5^b^	**0.003**
Thought problems	59.6 ± 7.2^a^	58.1 ± 7.0^a^	53.4 ± 5.2^b^	0.009
Attention problems	61.2 ± 8.0^a^	59.4 ± 7.6^a^	54.3 ± 6.1^b^	**0.004**
Total problems	63.1 ± 8.4^a^	60.5 ± 8.0^a^	54.2 ± 6.0^b^	**< 0.001**

Values are presented as mean ± SD. Overall group differences were analyzed using one-way analysis of variance (ANOVA). Because 11 CBCL/6–18 domain and broadband scores were tested, Bonferroni correction was applied, yielding a corrected significance threshold of p < 0.0045 (0.05/11 = 0.004545). For outcomes meeting this corrected threshold in the overall ANOVA, post-hoc pairwise comparisons were performed using Tukey’s honestly significant difference test. Groups sharing the same superscript letter are not significantly different, whereas groups with different superscript letters differ significantly in post-hoc comparisons. Bold values indicate statistically significant p-values.

In the multivariable linear regression analysis (see **Table 3**), offspring of parents with schizophrenia and offspring of parents with bipolar disorder had significantly higher CBCL/6–18 total problems T-scores than healthy controls, independent of age, sex, previous child psychiatry visit, and parental marital status. Compared with healthy controls, schizophrenia-group status was associated with a 7.94-point higher total problems score (B = 7.94, 95% CI: 3.71–12.17, p < 0.001), whereas bipolar-disorder-group status was associated with a 5.88-point higher score (B = 5.88, 95% CI: 1.76–10.00, p = 0.006). None of the covariates were significantly associated with the total problems score in the adjusted model.

**Table 3:** Multivariable linear regression analysis of factors associated with CBCL/6–18 total problems T-score.

Variable	B	SE	Standardized β	95% CI	p-value
Constant	49.82	4.91	—	39.98–59.66	< 0.001
Schizophrenia group (vs. healthy controls)	7.94	2.11	0.42	3.71–12.17	**< 0.001**
Bipolar disorder group (vs. healthy controls)	5.88	2.06	0.31	1.76–10.00	**0.006**
Age, years	0.21	0.24	0.08	−0.27–0.69	0.381
Male sex	0.67	1.74	0.03	−2.82–4.16	0.702
Previous child psychiatry visit	3.46	1.89	0.18	−0.33–7.25	0.072
Parental marital status (separated)	2.94	1.68	0.17	−0.43–6.31	0.085

Model statistics: R² = 0.39; Adjusted R² = 0.33; F = 6.37; p < 0.001.

B, unstandardized regression coefficient; SE, standard error; β, standardized regression coefficient; CI, confidence interval. Healthy controls were used as the reference category for the diagnostic group. Male sex was coded as 1 and female sex as 0. Separated parental marital status was coded as 1 and living together as 0.Bold values indicate statistically significant p-values.

## Discussion

The present study showed that children of parents with schizophrenia and children of parents with bipolar disorder had significantly higher CBCL/6–18 scores than healthy controls across a broad range of emotional and behavioral domains, including internalizing problems, externalizing problems, attention problems, social problems, thought problems, and total problems. Moreover, in the adjusted model, membership in both the schizophrenia and bipolar disorder offspring groups remained independently associated with higher total problems scores, suggesting that the observed elevation in psychopathology was not explained solely by basic demographic variables or selected family structure indicators. These findings support the view that familial risk for severe mental illness manifests not only as future disorder-specific liability but also as a broader and earlier vulnerability to dimensional psychopathology during childhood and adolescence (1, 3, 6).

Our findings are consistent with prior familial high-risk literature showing that offspring of parents with schizophrenia or bipolar disorder experience elevated emotional and behavioral difficulties from an early age. The Danish High Risk and Resilience Study demonstrated that 7-year-old children at familial high risk for schizophrenia spectrum psychosis or bipolar disorder had higher levels of psychopathology than controls across multiple dimensions (6, 17). Similarly, controlled and longitudinal studies have shown that offspring of parents with bipolar disorder display elevated rates of affective, anxiety, attentional, and disruptive symptoms, even before the typical age at onset of bipolar disorder (18–21). In parallel, offspring of parents with schizophrenia have been reported to show a broad range of childhood psychiatric difficulties, including impairments extending beyond narrowly defined psychotic-spectrum outcomes (11, 22, 23). Taken together, the present results add to a growing body of evidence that familial risk for schizophrenia and bipolar disorder is expressed in childhood as transdiagnostic psychopathology rather than as a purely syndrome-specific prodrome (1, 24).

An important feature of the current results is that the schizophrenia and bipolar disorder offspring groups were broadly similar across most CBCL/6–18 domains, while both differed from healthy controls. This pattern aligns with evidence that schizophrenia and bipolar disorder share part of their familial and developmental liability and that offspring of affected parents may exhibit overlapping early phenotypes (3, 24). The lack of substantial between-group differences in most symptom dimensions in our study may therefore reflect a genuinely shared early psychopathological burden across severe parental psychiatric disorders. A similar conclusion has emerged from transdiagnostic familial high-risk research showing that offspring vulnerability often crosses traditional diagnostic boundaries, with elevated risks for mood, anxiety, disruptive, attentional, and psychosis-related problems regardless of whether the parental disorder is schizophrenia or bipolar disorder (1, 8, 24).

At the same time, the descriptive pattern in our data suggests that the offspring of parents with schizophrenia tended to have numerically higher scores than the offspring of parents with bipolar disorder across nearly all CBCL/6–18 domains, although these differences were generally not statistically significant. This may indicate either a true but modest gradient of risk or limited statistical power to detect finer distinctions between the two clinical groups. Some previous studies have suggested that offspring of parents with schizophrenia may show greater abnormalities in domains related to cognitive organization, social functioning, or thought processes, whereas offspring of parents with bipolar disorder may show stronger affective lability and mood-related symptoms (11, 25, 26). Recent work has also reported somewhat differentiated symptom patterns between offspring of parents with schizophrenia and those of parents with bipolar disorder, although the overall burden of psychopathology remains elevated in both groups (27). In this context, our findings may be interpreted as supporting a primarily shared vulnerability model, with the possibility of subtler syndrome-linked differences that larger studies may be better able to detect.

The family-related characteristics observed in **Table 1** are also clinically relevant when interpreting the main findings. Compared with controls, the schizophrenia and bipolar disorder groups were characterized by higher rates of parental separation, lower income, lower parental educational attainment, lower employment rates, and higher rates of psychiatric disorder in the other parent. These differences are unlikely to be incidental, because prior research indicates that offspring outcomes in severe parental mental illness reflect the combined influence of genetic liability and environmental adversity (28–31). Family conflict, reduced cohesion, chronic stress exposure, impaired parental functioning, and social disadvantage have each been linked to worse outcomes in high-risk offspring, particularly among children of parents with bipolar disorder or psychotic disorders (30–32). Therefore, the elevated CBCL/6–18 scores observed in the present study should not be understood as reflecting inherited liability alone; rather, they likely represent the joint effects of familial vulnerability and adverse psychosocial context.

The multivariable regression findings reinforce the clinical relevance of the diagnostic grouping. Even after adjustment for age, sex, previous child psychiatry visit, and parental marital status, both schizophrenia-group status and bipolar-disorder-group status remained significantly associated with higher CBCL/6–18 total problems scores. This suggests that a parental diagnosis of severe mental illness provides information beyond a small set of background covariates and supports the use of parental diagnosis as an important marker for early developmental surveillance. These findings are in line with meta-analytic data showing that offspring of parents with severe mental illness are at increased risk not only for the same disorder as the parent but also for a wide range of other psychiatric outcomes, with one-third developing severe mental illness by early adulthood (1). Clinically, this argues for a low threshold for screening, psychoeducation, and family-oriented preventive care in these populations rather than a narrow watch-and-wait strategy focused only on psychosis or bipolar conversion (33, 34).

From a practice perspective, our results support the value of early identification in children of parents with schizophrenia or bipolar disorder. The pattern of elevated internalizing, externalizing, attentional, social, and overall problem scores suggests that these children may come to clinical attention long before any disorder-specific adult phenotype emerges. This has practical implications for routine psychiatric care of affected parents: clinicians should ask about offspring functioning, family stressors, school difficulties, and prior mental health contacts, and should consider referral pathways for child mental health assessment when appropriate. Family-focused preventive approaches have shown promise in improving child and family outcomes in the context of severe parental mental illness, underscoring that this risk is potentially modifiable and not merely deterministic.

This study has several limitations. First, its cross-sectional design precludes causal inferences and does not allow evaluation of developmental trajectories over time. Second, the sample size was modest and the study was conducted at a single center, which may limit statistical power for detecting subtle differences between the schizophrenia and bipolar disorder offspring groups and may reduce generalizability. Third, behavioral and emotional problems were assessed primarily through parent-report CBCL/6–18 data, which may be influenced by informant-related bias. Fourth, because of the modest sample size, the regression model included a limited number of covariates, and residual confounding by socioeconomic and family-related factors cannot be excluded. Finally, the clinical groups differed from controls in several family-related and socioeconomic characteristics, and residual confounding by unmeasured environmental factors cannot be excluded.

## Conclusion

In conclusion, this study found that children of parents with schizophrenia and children of parents with bipolar disorder had significantly higher emotional and behavioral problem scores than healthy controls across multiple CBCL/6–18 domains. The findings support a transdiagnostic model of early vulnerability in offspring of parents with severe mental illness, with elevated difficulties in internalizing, externalizing, attentional, social, thought-related, and overall problem dimensions. Even after adjustment for selected covariates, both schizophrenia-group status and bipolar-disorder-group status remained independently associated with higher total problems scores.

These results highlight the importance of systematic child-focused screening in routine adult psychiatric care and support early, family-oriented preventive strategies for offspring of parents with schizophrenia or bipolar disorder. Larger longitudinal studies using multi-informant and diagnostically standardized assessments are needed to clarify developmental trajectories, identify disorder-specific versus shared risk patterns, and determine which psychosocial and clinical factors are most amenable to intervention.

## Data Availability

The datasets generated and/or analyzed during the current study are available from the corresponding author upon reasonable request.
